# Scoring of swine lung images: a comparison between a computer vision system and human evaluators

**DOI:** 10.1186/s13567-024-01432-5

**Published:** 2025-01-13

**Authors:** Robert Valeris-Chacin, Beatriz Garcia-Morante, Marina Sibila, Albert Canturri, Isaac Ballarà Rodriguez, Ignacio Bernal Orozco, Ramon Jordà Casadevall, Pedro Muñoz, Maria Pieters

**Affiliations:** 1 Veterinary Education, Research, and Outreach (VERO), Department of Veterinary Pathobiology, College of Veterinary Medicine and Biomedical Sciences, Texas A&M University, Canyon, TX USA; 2 Unitat Mixta d’Investigació IRTA-UAB en Sanitat Animal, Centre de Recerca en Sanitat Animal (CReSA), Campus de La Universitat Autònoma de Barcelona (UAB), 08193 Bellaterra, Catalonia Spain; 3 IRTA, Programa de Sanitat Animal, Centre de Recerca en Sanitat Animal (CReSA), Campus de la Universitat Autònoma de Barcelona (UAB), 08193 Bellaterra, Catalonia Spain; 4 WOAH Collaborating Centre for the Research and Control of Emerging and Re-Emerging Swine Diseases in Europe (IRTA-CReSA), 08193 Barcelona, Bellaterra Spain; 5 Veterinary Diagnostic Laboratory, College of Veterinary Medicine, University of Minnesota, St. Paul, MN USA; 6 Department of Veterinary Population Medicine, College of Veterinary Medicine, University of Minnesota, St. Paul, MN USA; 7 LABORATORIOS HIPRA, S.A, 17170 Girona, Amer Spain; 8 Swine Disease Eradication Center, College of Veterinary Medicine, University of Minnesota, St. Paul, MN USA

**Keywords:** Artificial intelligence, algorithm, *Mycoplasma hyopneumoniae*, cranioventral pulmonary consolidation, lung, lesions, slaughterhouse, pigs

## Abstract

Cranioventral pulmonary consolidation (CVPC) is a common lesion observed in the lungs of slaughtered pigs, often associated with *Mycoplasma* (*M.*) *hyopneumoniae* infection. There is a need to implement simple, fast, and valid CVPC scoring methods. Therefore, this study aimed to compare CVPC scores provided by a computer vision system (CVS; AI DIAGNOS) from lung images obtained at slaughter, with scores assigned by human evaluators. In addition, intra- and inter-evaluator variability were assessed and compared to intra-CVS variability. A total of 1050 dorsal view images of swine lungs were analyzed. Total lung lesion score, lesion score per lung lobe, and percentage of affected lung area were employed as outcomes for the evaluation. The CVS showed moderate accuracy (62–71%) in discriminating between non-lesioned and lesioned lung lobes in all but the diaphragmatic lobes. A low multiclass classification accuracy at the lung lobe level (24–36%) was observed. A moderate to high inter-evaluator variability was noticed depending on the lung lobe, as shown by the intraclass correlation coefficient (ICC: 0.29–0.6). The intra-evaluator variability was low and similar among the different outcomes and lung lobes, although the observed ICC slightly differed among evaluators. In contrast, the CVS scoring was identical per lobe per image. The results of this study suggest that the CVS AI DIAGNOS could be used as an alternative to the manual scoring of CVPC during slaughter inspections due to its accuracy in binary classification and its perfect consistency in the scoring.

**Supplementary Information:**

The online version contains supplementary material available at 10.1186/s13567-024-01432-5.

## Introduction

Respiratory diseases are one of the most important health issues associated with growing/finishing pigs, as they affect animal wellbeing and increase production costs [1, 2]. Moreover, respiratory diseases of bacterial origin remain one of the main reasons for the prescription of antibiotic treatments in pigs [3, 4]. One type of respiratory disorders, pneumonia, can be observed in pigs at the slaughterhouse, with a reported prevalence ranging from 8.4% to 73.1%, depending on various factors, such as country, region, production system and vaccination status against respiratory pathogens, among others [2, 5–9].

Cranioventral pulmonary consolidation (CVPC; [6]) is a common macroscopic morphologic pneumonic pattern observed in swine *post mortem* and is often associated with *Mycoplasma* (*M.*) *hyopneumoniae* infection [2, 5, 6, 8, 9]. Infections with *M. hyopneumoniae* are highly prevalent worldwide and predispose pigs to other respiratory pathogens, leading to chronic or polymicrobial diseases, namely enzootic pneumonia or the porcine respiratory disease complex [10]. The degree of CVPC associated with *M. hyopneumoniae* infection can be scored and recorded at the slaughterhouse to estimate disease prevalence and extension, as well as the impact of implemented prevention and control strategies [11]. Several scoring methods based on direct macroscopic observation of lung tissue have been developed to assess CVPC in farmed pigs (reviewed in [12, 13]). Current CVPC scoring methods are considered subjective and not fully reliable, as lung lesion evaluation may in some cases vary within and between evaluators [14–18]. In addition, lung lesion scoring can be a time-consuming and expensive activity, requiring the physical location of the evaluator at the slaughterhouse, at the specific time when a pig batch is processed. Thus, the development and implementation of simple, fast, valid, and easily standardisable lung lesions scoring approaches for reliable and consistent data output are highly desirable.

Recent progress in the field of artificial intelligence (AI) has allowed the development of high-performance algorithms introduced to the veterinary image analysis domain [19–25]. In pigs, computer vision systems (CVS) based on machine learning algorithms have been shown to identify and score pneumonia and pleurisy from digital images captured at slaughterhouses with high accuracy when compared with human operators [26–28]. However, there is a lack of information about common situations during the deployment of any CSV using CVPC scoring, such as comparing the CVS scoring with that of evaluators not involved in the system training and in the context of moderate inter-evaluator variability. Therefore, this study aimed to compare the CVPC scores on swine lung images assigned by a prototype CVS with the scores assigned by human evaluators. Additionally, the variability in the CVPC scoring between and within the evaluators, and within the CVS, was investigated.

## Materials and methods

### Computer vision system

**Figure 1:**
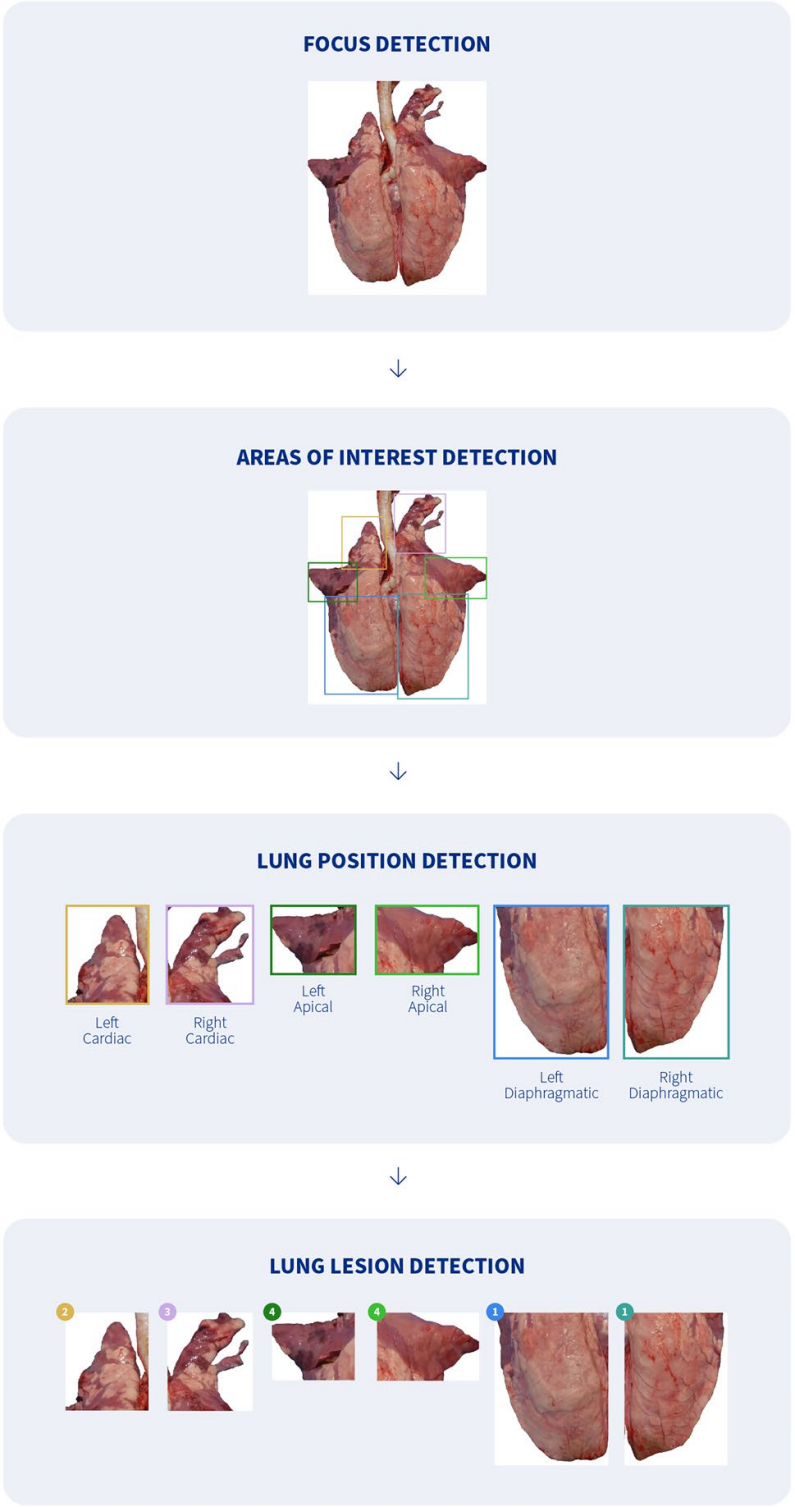
**Graphic representation of the image analysis process comprising the computer vision system.** The squares indicate the area of lung detection by the software, different colors identifying each lobe. The predicted score of each lobe is shown in a circle with the same color as the corresponding lung lobe.

A CVS developed by HIPRA Laboratories (Girona, Spain), named AI DIAGNOS, was used to recognize and score CVPC on digital images captured from pig lungs at slaughter [29]. The CVS is an Amazon SageMaker^™^ that works on two core processes; detection and classification. The general processes of detection and classification are shown in Figure 1. In brief, a lung image first passes through a filter (focus detector) with the aim of differentiating the lung from the background. Subsequently, the processed image is passed through an area of interest detector that identifies each of the lung lobes visible from a dorsal view (i.e., the accessory lobe is not evaluated). A new filter is then applied to each lung lobe to check the position of the lung within the image and to correct possible detection errors. Thereafter, each lung lobe is cropped and saved separately, maintaining the relationship with the initial lung image. Finally, each lung lobe is passed through a final filter (Convolutional Neural Network Classifier, RestNet18), which is trained to predict the lesion score of each lung lobe based on the lung lesion scoring method referred to as Madec and Kobisch [30], which is commonly employed to evaluate CVPC.

HIPRA Laboratories conducted the training of the CVS with 13 864 images scored by a total of six evaluators. Two from North America and four from Europe. Evaluators were eligible to participate in the CVS training based on demonstrated expertise in the scoring of swine lung lesions associated with *M. hyopneumoniae* infection (> 5 years evaluating lung lesions associated with natural and/or experimental *M. hyopneumoniae* infection). An accuracy of 85% was reported for this CVS during training [29]. After the training was concluded, new images were collected and the evaluation of the CVS was performed (reported in this study). Five evaluators (A-E) participated in the evaluation phase. Four out of these five evaluators also participated in the training phase, in total scoring 35% of the lung images of the training set.

### Source of lung images and allocation for scoring

**Figure 2:**
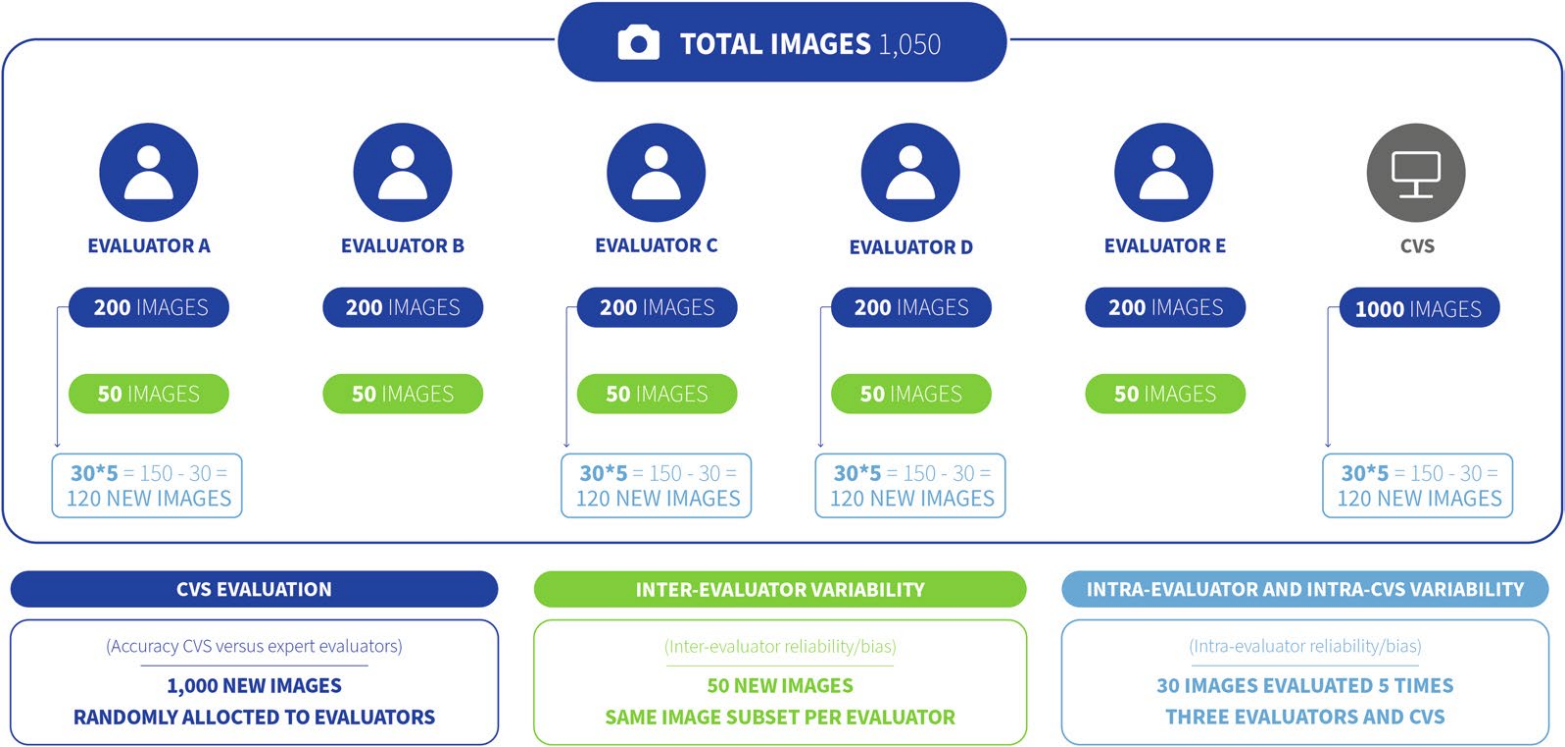
**Graphic representation of the entire dataset used for computer vision system (CVS) evaluation (dark blue), inter-evaluator variability (green), and intra-evaluator and intra-CVS variability (light blue).** A total of 1000 images were used to evaluate the CVS accuracy. A set of 50 extra images served to estimate the inter-evaluator variability. Four sets of 30 images used for CVS evaluation were randomly selected to assess the intra-evaluator and intra-CVS variability (three sets and one set, respectively). Each set only required the duplication of the original images four times, adding 120 more images per set. In total, the images in those sets were evaluated five times to assess the intra-evaluator and intra-CVS variability.

A total of 1050 dorsal view images of swine lungs were randomly selected from a larger pool taken at a slaughterhouse in Spain. The images were collected in an area duly separated from the slaughter line (right after the separation of lungs from the heart) and obtaining approval from the slaughterhouse administration. Lungs were placed on a metallic table and one dorsal image per lung was taken with a Samsung smartphone (model SM-A21F) with the following settings: RGB color space, focal length of 4.6 mm, center-weighted average metering mode, F number of f/2, and exposure time of 1/50. Images were obtained from January to June of 2021, and were randomly assigned to the following analyses (Figure 2):

1. CVS evaluation: 1000 images were used to evaluate the accuracy of the CVS. The images were randomly assigned to five evaluators (A-E), each having a set of 200. The scores given by the evaluators were used as the true score. The scores produced by the CVS were then compared to those of the evaluators. This sample size would allow the estimation of a misclassification error of 26.1% (or lower) assuming an overall accuracy of 70%, power of 80%, and 5% of significance level (power oneproportion function in Stata 17 [31]).
2. Inter-evaluator variability: A set of 50 new images was assigned to all evaluators to estimate the inter-evaluator variability. This sample size would allow the detection of an intraclass correlation (ICC) of 0.7 (or greater), considering an acceptable ICC of 0.5 (the null hypothesis), a significance level of 0.05, and a power of 80% [32].
3. Intra-evaluator and intra-CVS variability: Three sets of 30 images, randomly selected from the sets used for the CVS evaluation (*n* = 1000), were used to measure the variability within three evaluators (one set per evaluator). Each image was scored five times by the corresponding evaluator. Additionally, another 30-image set was randomly selected to assess the variability within the CVS. The random selection of the 30-image sets was conducted independently from each other. This sample size would allow the detection of an ICC of 0.95 (or greater), considering an acceptable ICC of 0.9 (the null hypothesis), a significance level of 0.05, and a power of 80% [32].

In total, two evaluators scored 250 lung images, whereas three evaluators scored 370. One thousand one hundred and twenty images were scored by the CVS. Images were de-identified and their order was randomly shuffled prior to scoring by the evaluators. Access to images was individually assigned to each evaluator in the form of a Google sheet containing hyperlinks to each de-identified image, plus labelled columns (one per lung lobe) for adding the score.

### Lung lesion scoring by evaluators

The evaluators applied the same lung lesion scoring method as the CVS  (Madec and Kobisch) [30] to assign scores to individual lobes in the dorsal view of lung images. The method divides each lobe into quarters and scores, each affected quarter with one point. Thus, the minimum score is zero (lung lobe shows no lesion), and the maximum is four (at least 75% of lung lobe is affected). The accessory lung lobe was not observable in the images, and therefore, was not evaluated. Consequently, the maximum total lung lesion score could be 24 instead of 28. Moreover, in the case that any lung lobe could not be scored due to incomplete visualization or lack of a good quality image, the written abbreviation for not applicable “NA” was recorded. Evaluators were masked to the scoring performed by the CVS and other evaluators.

In addition, the percentage of affected lung area (%LA) was calculated by multiplying the Madec and Kobisch score per lung lobe by a weight reflecting the area of that lobe in the total lung according to Christensen, Sorensen, and Mousing [33], adjusting for the accessory lung lobe, which was not evaluated. Hence, the weights were as follows: 0.06 for left apical lung lobe, 0.11 for right apical lung lobe, 0.07 for left cardiac lung lobe, 0.12 for right cardiac lung lobe, 0.29 for left diaphragmatic lung lobe, and 0.35 for right diaphragmatic lung lobe. The final value was multiplied by 25 to transform it to a percentage.

### Lung lesion scoring by the computer vision system

All lung images selected for the CVS evaluation were uploaded to the system. Lung images were uploaded in batches of 250 to guarantee the proper analysis by all the components of the CVS. Image batches were independent of the assignment to evaluators.

### Statistical analysis

The preprocessing of data and the building of the models to evaluate the different types of variability were performed in Stata 17 [31]. The statistical significance level was set a priori at 0.05.

The evaluation of the CVS for lung scoring was undertaken with three different metrics per lung lobe: balanced accuracy (the average of the sensitivity for each class) [34], chance-adjusted balanced accuracy (e.g., a random classifier will have a score of 0) [34], and macro ROC area under curve (AUC) for multiclass classification (summary of the predictive power of the CVS averaging the class-specific AUC values) [35] in Python 3.8.13 and scikit-learn 1.0.2. The one-versus-rest approach was selected for the calculation of the macro ROC AUC (i.e., computing the ROC AUC for each class against the rest) [35]. The scores provided by the evaluators were considered the true status. Lung scores from the evaluators and CVS were also dichotomized (0 = score of 0; 1 = score > 0) per lung lobe and overall. The dichotomized scores were utilized in the calculation of balanced accuracy, adjusted balanced accuracy, and ROC AUC. The correlation coefficient between the %LA estimated from the evaluator scoring and that from the CVS, was calculated.

In order to evaluate the inter-evaluator variability, both binary and multiclass accuracies were calculated between each pair of evaluators. The inter-evaluator variability was also assessed by calculating the intra-class correlation (ICC) in ANOVA models with two random effects [36, 37]: lung image identification (ID) and evaluator ID. Separate ICC models were built for the lung lesion scores (total and per lung lobe) and %LA.

The same outcomes described above were utilized in the estimation of the intra-evaluator variability via the ICC from two-way mixed-effects ANOVA models [36, 37], in which lung image ID and order were considered random and fixed effects, respectively. Separate models were created for each of the three randomly selected evaluators. As for the intra-CVS variability, ICC values were calculated from one-way random-effects ANOVA models [36, 37] built for lung lesion scores (total and per lung lobe) and %LA with lung image ID as the random effect.

## Results

Four percent of the lung lobe scorings (238/6000) performed by the evaluators were missing due to incomplete visualization or blurry image. The proportion of missingness was not associated to any specific evaluator (Fisher’s exact test, *p*-value = 0.159), nor with the lung score estimated by the CVS (Fisher’s exact test, *p*-value = 0.2; Additional file 1). Missingness was the highest in the right apical lobe (9.1%) and the lowest in the right diaphragmatic lobe (1%).

**Figure 3:**
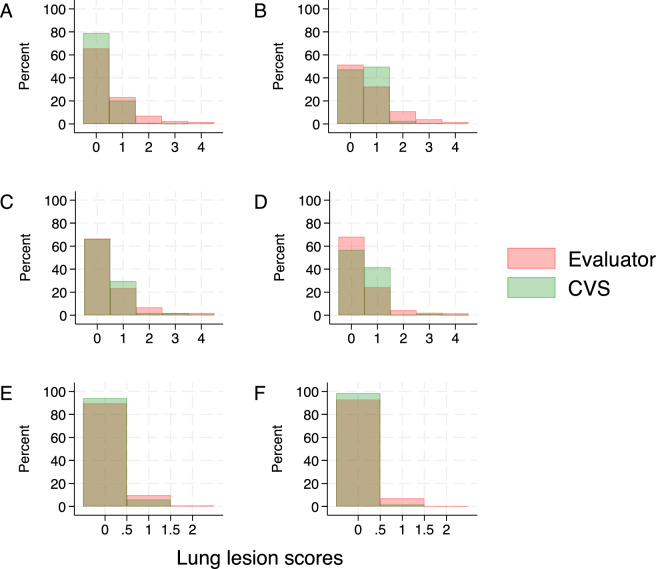
**Distribution of lung lesion scores in lung lobes. Histograms of the scores by the evaluators and the computer vision system (CVS) are color-coded and overlaid.** Data for individual lung lobes are shown in different panels: **A** left apical; **B** right apical; **C** left cardiac; **D** right cardiac; **E** left diaphragmatic; **F** right diaphragmatic.

The distribution of the lung scores from the evaluators and CVS per lung lobe is shown in Figure 3. Except for the right cardiac lobe, the lung scorings performed by the CVS were, on average, significantly lower than those from the evaluators (paired t tests, *p*-value < 0.005).

### Computer Vision System evaluation

**Figure 4:**
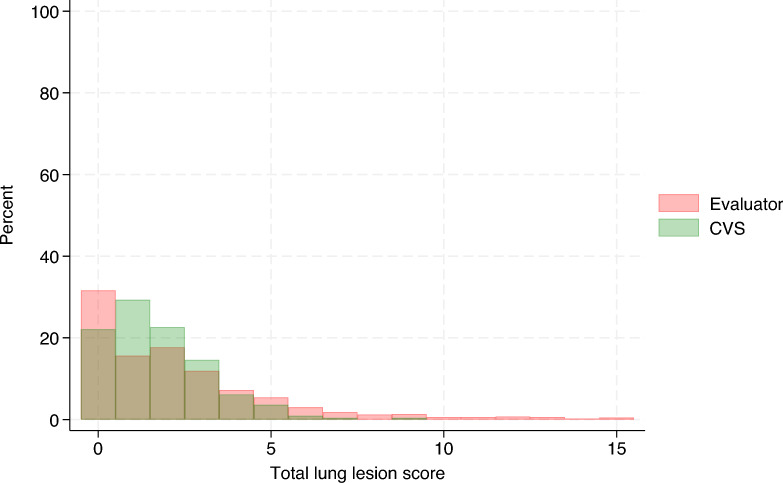
**Distribution of the total lung lesion scores**. Histograms of the lung lesion scores from evaluators and the computer vision system (CVS) are color-coded and overlaid.

**Figure 5:**
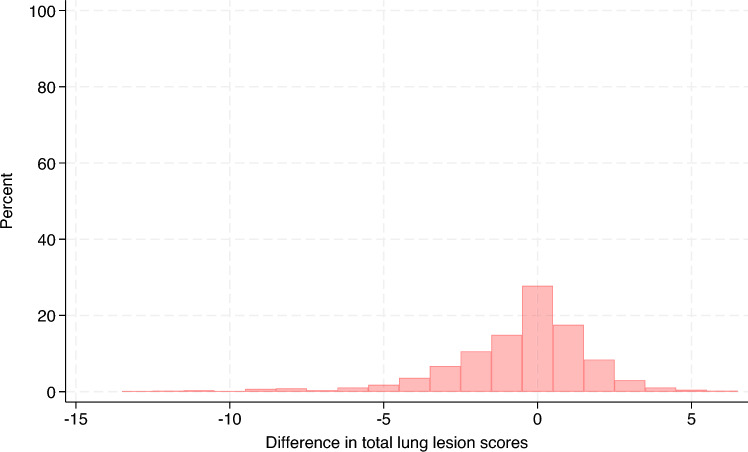
**Distribution of the difference in total lung lesion scores between the computer vision system (CVS) and evaluators.** Scores from the evaluators were subtracted from the corresponding CVS scores to calculate the differences.

**Figure 6:**
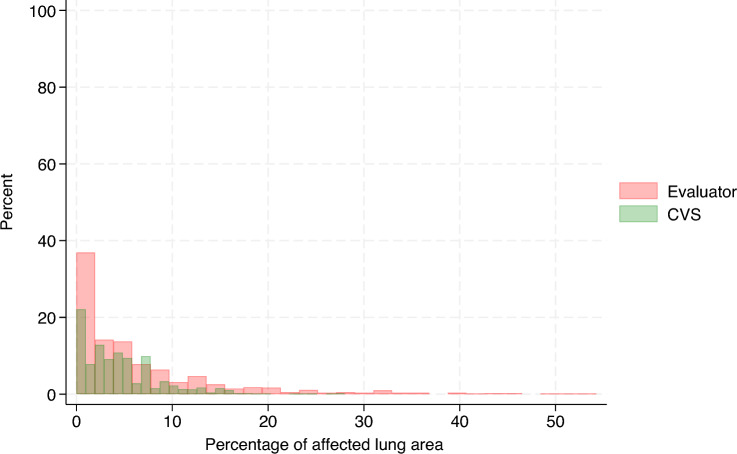
**Distribution of the percentage of affected lung area**. Histograms of the percentage of affected lung area from all evaluators and the computer vision system (CVS) are color-coded and overlaid.

**Figure 7:**
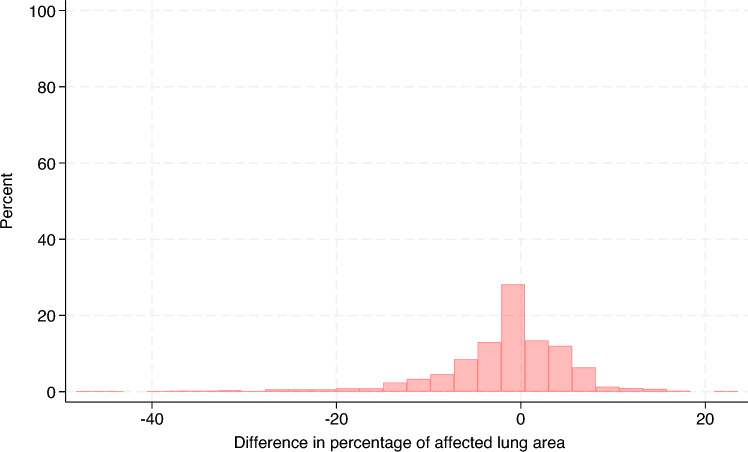
**Distribution of the difference in the percentage of affected lung area between the computer vision system (CVS) and evaluators.** The percentage of affected lung area from the evaluators was subtracted from the corresponding CVS value to calculate the difference.

Table 1 summarizes the evaluation of the CVS, which showed low balanced accuracy (range = 0.24–0.36) in the multiclass classification setting (see Additional files 1–3). Conversely, in the binary classification setting, the CVS achieved moderate balanced accuracy (range = 0.62–0.71) in all lung lobes except for the diaphragmatic left and right (see Additional files 4–6). The distribution of the total lung lesion scores from the evaluators and those from the CVS is shown in Figure 4 and the distribution of their differences (25% percentile = −2, median = 0, 75% percentile = 1) is displayed in Figure 5. On average, the total lung lesion scores obtained from the CVS were significantly lower than the scores from the evaluators (mean difference = −0.6; 95% CI  −0.76, −0.44; paired t test, *p*-value < 0.0001). A moderate correlation coefficient was observed between the total lung lesion scores from the evaluators and those from the CVS (correlation coefficient = 0.5; percentile boostrapped 95% CI 0.44, 0.55). The distribution of the %LA estimated from the evaluator scoring and that from the CVS is shown in Figure 6 and the distribution of their difference (25% percentile = −4.25%, median = 0%, 75% percentile = 2.75%) is displayed in Figure 7. The same general trend as with the lung lesion scores per lung lobe and total can be observed, i.e., the mean %LA derived from the CVS scoring is significantly lower than that from the evaluators (mean difference = -1.8%; 95% CI −2.3%, −1.3%; paired t-test, *p*-value < 0.0001). The corresponding correlation coefficient was 0.43 (percentile bootstrapped 95% CI 0.36, 0.5). A moderate accuracy (ROC = 0.63, balanced accuracy = 0.63, adjusted balanced accuracy = 0.27) was also observed when the total lung lesion scores were dichotomized (lungs with or without lesions).

**Table 1:** **Evaluation of the lung lesion scoring by a computer vision system.**

Lung lobe	Multiclass classification			Binary classification		
	ROC AUC (macro)	Balanced accuracy	Adjusted balanced accuracy	ROC AUC	Balanced accuracy	Adjusted balanced accuracy
LA	0.54	0.24	0.05	0.62	0.62	0.25
RA	0.58	0.29	0.11	0.71	0.71	0.41
LC	0.59	0.31	0.14	0.69	0.69	0.39
RC	0.58	0.3	0.13	0.67	0.67	0.33
LD	0.53	0.36	0.04	0.54	0.54	0.08
RD	0.52	0.35	0.03	0.53	0.53	0.06

The lung lesion scoring by human evaluators was considered the true status.

LA: left apical, RA: right apical, LC: left cardiac, RC: right cardiac, LD: left diaphragmatic, RD: right diaphragmatic, ROC AUC: Receiver Operating Characteristic Area Under the Curve.

### Inter-evaluator variability

A moderate inter-evaluator variability was observed in this study (Table 2) with high heterogeneity of the ICC values based on the lung lobe. A wide distribution of the balanced accuracy between the pairs of evaluators in both the binary and multiclass settings (see Additional files 7 and 8) was evidenced. High heterogeneity was also observed in the agreement on missingness among the evaluators. There was a perfect agreement in the right diaphragmatic lobe, followed by the left apical lobe (kappa = 0.86, *p*-value < 0.0001), and the right cardiac lobe (kappa = 0.49, *p*-value < 0.0001). The missingness in the rest of the lung lobes showed very low agreement among evaluators.

**Table 2:** **Inter-evaluator variability on lung lesion scoring**.

	ICC	95% CI	*P* value
Total lung lesion score	0.52	(0.35, 0.68)	< 0.001
Lesion score per lung lobe			
LA	0.29	(0.16, 0.45)	< 0.001
RA	0.42	(0.25, 0.59)	< 0.001
LC	0.43	(0.3, 0.58)	< 0.001
RC	0.6	(0.47, 0.72)	< 0.001
LD	0.43	(0.3, 0.57)	< 0.001
RD	0.34	(0.21, 0.48)	< 0.001
%LA	0.57	(0.42, 0.72)	< 0.001

ICC: intra-class correlation coefficient (1 indicates perfect agreement between evaluators), LA: left apical, RA: right apical, LC: left cardiac, RC: right cardiac, LD: left diaphragmatic, RD: right diaphragmatic, %LA: percentage of affected lung area.

### Intra-evaluator and intra-CVS variability

The intra-evaluator variability was low and similar among the different metrics and lung lobes (Table 3). The lowest ICC values were obtained in the right diaphragmatic lobe. The observed ICC values differed among evaluators. By contrast, the CVS scoring was identical per lobe per image, obtaining an ICC of 1 in all metrics (Table 3).

**Table 3:** **Intra-evaluator and intra-computer vision system variability on lung lesion scoring.**

	Evaluator A	Evaluator C	Evaluator D	CVS
Total lung lesion score	0.96 (0.93, 0.98)	0.99 (0.98, 1)	0.86 (0.76, 0.93)	1 (n/e)
Lesion score per lung lobe				
LA	0.91 (0.85, 0.95)	0.98 (0.97, 0.99)	0.79 (0.66, 0.88)	1 (n/e)
RA	0.9 (0.83, 0.95)	1 (n/e)	0.73 (0.59, 0.84)	1 (n/e)
LC	0.96 (0.94, 0.98)	0.97 (0.94, 0.98)	0.82 (0.71, 0.90)	1 (n/e)
RC	0.94 (0.91, 0.97)	0.95 (0.92, 0.97)	0.76 (0.64, 0.86)	1 (n/e)
LD	0.66 (0.51, 0.79)	1 (n/e)	0.92 (0.87, 0.96)	1 (n/e)
RD	n/e*	0.86 (0.78, 0.92)	0.68 (0.53, 0.81)	1 (n/e)
%LA	0.95 (0.91, 0.98)	0.98 (0.96, 0.99)	0.87 (0.78, 0.93)	1 (n/e)

Values represent the intra-class correlation coefficients. 95% Confidence Intervals within parentheses.

LA: left apical, RA: right apical, LC: left cardiac, RC: right cardiac, LD: left diaphragmatic, RD: right diaphragmatic, %LA: percentage of affected lung area.

^*^n/e: not estimable due to lack of variability in the lung lesion scores.

## Discussion

This study interrogated the accuracy of a CVS trained to assign CVPC scores from lung images obtained at slaughter, while assessing the variability between and within human evaluators, and within the CVS on the lung lesion scoring. The results of this study showed that the CVS was able to discriminate lung lobes affected with CVPC from non-affected ones, with moderate accuracy. However, the CVS ability to differentiate CVPC-affected from non-lesioned was highly variable between lung lobes. Additionally, the CVS ability to correctly assign the extension of pneumonia at the lung lobe level was low. Remarkably, the CVS showed a perfect repeatability in this study.

Two CVS in pigs for detecting and scoring respiratory lesions caused by infectious agents have been developed and tested [26–28]. These systems were developed to automatically score pleurisy associated to *Actinobacillus pleuropneumoniae* [28], and to detect and measure CVPC in the slaughter processing line [26]. The average multiclass-classification accuracy for pleurisy was 85.5% [28], while the average specificity and sensitivity for the binary-classification of CVPC were, respectively, 95.31% and 99.38% [26]. In a more recent evaluation of the CVS for CVPC scoring in the slaughter processing line, very high specificity (95.55%) and high sensitivity (85.05%) were observed when the CVS scoring was compared with a skilled operator inspecting the lungs via visual inspection and palpation [27]. The rather low multiclass classification performance estimated for the CVS could have been influenced by one or more of the following factors: imbalanced training data set, the presence of artifacts, and a moderate inter-evaluator variability. A lower proportion of lung lobes scoring 2, 3 and 4 compared with those scoring 0 and 1 were used to train the CVS. Indeed, the scorings performed by the evaluators were, in general, significantly associated with higher lung lesion scoring than the CVS. While the balanced accuracy was used herein to minimize the bias due to the imbalanced data set, this could be further addressed by increasing the number of observations in the training set, thus progressively improving the CVS performance.

The presence of artifacts (e.g., blood inspiration, atelectasis due to mechanical compression, damaged or folded lung lobes) or scarring due to resolved CVPC, not easily interpreted even by human evaluators, could have potentially affected data quality. In fact, lungs entirely filled with blood or severely torn due to chronic pleurisy were not included in the CVS validation developed by [26]. In this context, the CVS assessed in this study could offer significant benefits by delivering enhanced value with consistent results in diverse slaughterhouse conditions.

The reported accuracy could be conceived as an average of the agreements between the CVS and each human evaluator. Hence, the variability observed between evaluators could have had a negative impact on the CVS accuracy. It is worth mentioning that images were annotated by two evaluators in the abovementioned previous works [26, 28], whereas five evaluators participated in this study, adding greater variability in the CVS performance assessment.

Pathological findings for which there are different levels of gradation to choose from, as pneumonia, typically exhibit larger variation among meat inspectors [18]. Even though the evaluators in this study were experts in assessing CVPC from pig lungs, a moderate to high inter-evaluator variability was observed depending on the lung lobe (i.e., ICC ranging 0.29–0.6), which agrees with results reported in other studies. For instance, the agreement among results concerning 20 plucks given by 11 veterinarians in Germany using Kendall's coefficient of concordance was examined [17]. The results for lung lesions showed a low degree of agreement (25%) among the meat inspectors. The detection rates of 12 meat inspectors using the variance partitioning coefficient (VPC) were compared [18] and a moderate VPC for pneumonia (0.68–2.32%) was determined. Indeed, in that study broad ranges of mild (3.2–26.8%), moderate (4.8–23.5%) and severe (1.5–16.2%) estimated pneumonia prevalence were reported. Moderate variations in the observation of pneumonia and pleuritis have been also documented in other studies [15, 16]. Even though the CVPC scoring used in this study is not routinely performed during meat inspection, these previously reported results and our findings highlight the substantial variations between evaluators in scoring CVPC extension, probably due to systematic differences, as well as variations in anatomic-pathologic definitions.

The lack of a standardized meat inspection process is also posed as one of the main causes of the low intra-observer reliability sometimes observed [16, 18, 38]. In this study, the overall intra-evaluator reliability estimates were in general very high, although they slightly differed among evaluators (i.e., ICC ranging 0.89–0.99). Hence, the results indicate an overall high level of consistency within their own scoring process of CVPC, which can be a reflection of their expertise. Remarkably, the CVS scoring was identical per lobe per image, showing perfect consistency. This capability has the potential of effectively reducing the inherent variability that exists between and within human evaluators, addressing a crucial issue in current scoring methods.

One potential limitation of this study is that images, either evaluated by a human or by a CVS, cannot convey the tactile element of the macroscopic assessment. The concept of CVPC is tactile in essence, based on an increased consistency of the pulmonary parenchyma that is noted by pressing with the fingers and not by visual inspection alone [39]. In the study by Bonicelli et al. [26], all veterinarians involved in photo annotations could palpate the lungs to confirm/rule out CVPC if necessary, which was not feasible in the present work. Interestingly, a strong positive correlation (0.81) has been observed between the Madec and Kobisch scores (visual and palpation) and the Blaha scores (visual only) performed by two operators in an Italian high-throughput slaughterhouse, with discrepancies concentrated on the discrimination between healthy lungs and those with minor lesions [40]. Another limitation of the present CVS and the one developed by Bonicelli et al. [26] is that the ventral view of the lung was not assessed, which may underestimate the overall CVPC severity and extension. Notably, an image analysis software to score *M. hyopneumoniae*-associated lung lesions on digital images under experimental conditions was used by Garcia-Morante et al. [12]. Such system correlated with other conventional scoring methods, although lesions of the accessory lobe were not accounted for and partially affected the Pearson’s coefficient [12]. Thus, simplified slaughter check procedures have been suggested (e.g., based on the examination of a single lung view or half carcass) to ease the collection of images and to further improve the efficiency of the scoring method and the implementation in the slaughter line [41].

The use of AI methods has revolutionized many different aspects of health sciences, especially by enhancing our capabilities to extract quantitative information from digital images that can then be used to predict the presence of lesions in digitized glass slides [42], radiographs [43], or ultrasound images [24]. To date, few CVS have been developed to monitor slaughter lesions in pigs [26–28, 44, 45]. The prototype CVS employed herein is conceived as a fully automatic method for systematic detection and scoring of CVPC. The CVS delivers a well-known and broadly used lung scoring method in pigs [30]. It also calculates results in reference to the scheme reported by Christensen, Sorensen, and Mousing [33], thus providing additional value by reporting the total weighted percentage of affected lung. Future studies are expected to refine and improve the accuracy and efficiency of the CVS in detecting and scoring CVPC, granting invaluable support to the swine industry.

In conclusion, under the conditions of this study, the CVS (AI DIAGNOS) discriminated between pneumonic and non-pneumonic lung lobes with moderate accuracy, suggesting that this CVS could be a good alternative to the detection of CVPC during slaughter inspections, especially considering its perfect consistency in the lung lesion scoring compared with the human evaluators. This prototype CVS will be further refined to correctly predict the degree of pneumonia. Altogether, the CVS evaluated in this study has the potential to automatically score *M. hyopneumoniae*-associated lung lesions and facilitate processing of data to aid solving problems linked to conventional CVPC scoring at slaughter.

## Supplementary Information

**Additional file 1: Multiclass accuracy for the computer vision system in the left and right apical lobes.** Classes are based on Madec and Kobisch [30].

**Additional file 2: Multiclass accuracy for the computer vision system in the left and right cardiac lobes.** Classes are based on Madec and Kobisch [30]

**Additional file 3: Multiclass accuracy for the computer vision system in the left and right diaphragmatic lobes.** Classes are based on Madec and Kobisch [30].

**Additional file 4: Binary accuracy for the computer vision system in the left and right apical lobes.**

**Additional file 5: Binary accuracy for the computer vision system in the left and right cardiac lobes.**

**Additional file 6: Binary accuracy for the computer vision system in the left and right diaphragmatic lobes.**

**Additional file 7: Distribution of the balanced accuracy between pairs of evaluators in the binary setting**. Data is shown for the left and right apical lobes.

**Additional file 8: Distribution of the balanced accuracy between pairs of evaluators in the multiclass setting**. Data is shown for the left and right apical lobes. Classes are based on Madec and Kobisch [30].

## Data Availability

All data generated or analysed during this study can be available from the corresponding author upon request.
