# Supplementary figures and images for: Scoring of swine lung images: a comparison between a computer vision system and human evaluators
Source: Vet Res. 2025 Jan 13;56:9. doi: 10.1186/s13567-024-01432-5 (PMC11731141; doi:10.1186/s13567-024-01432-5)

Multiclass accuracy in left apical lobe

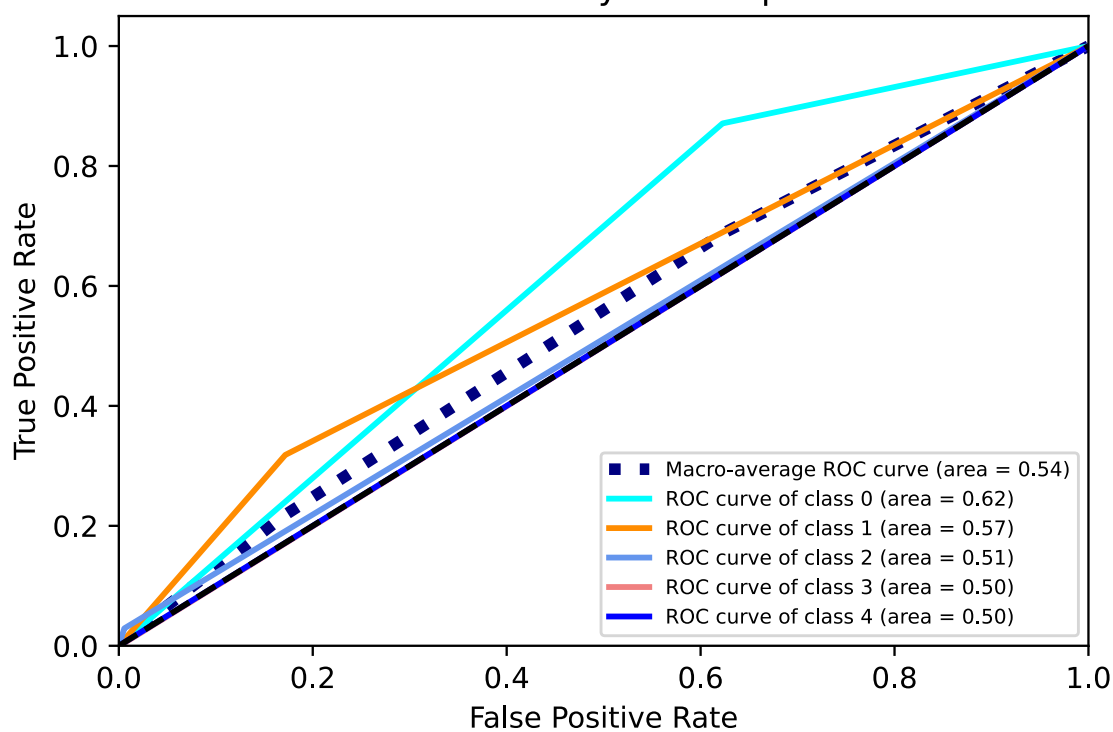

Multiclass accuracy in right apical lobe

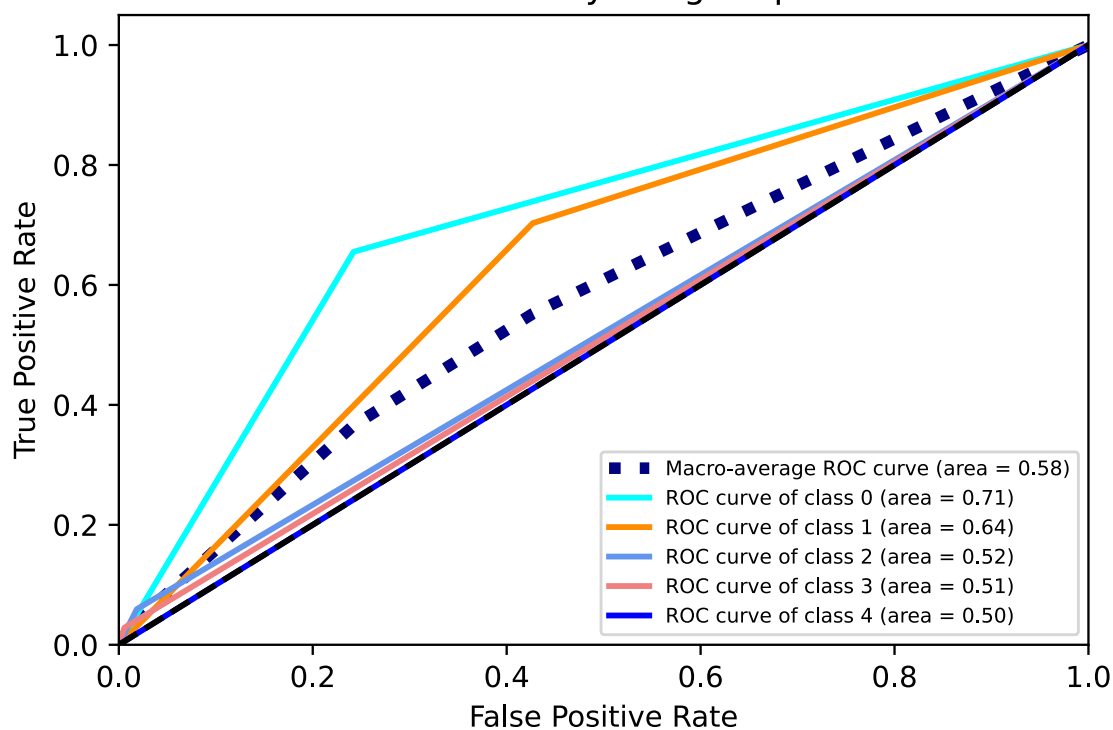

Supplement: Supplementary file 1 — Additional file 1: Multiclass accuracy for the computer vision system in the left and right apical lobes. Classes are based on Madec and Kobisch [30]. [file 13567_2024_1432_MOESM1_ESM.pdf]

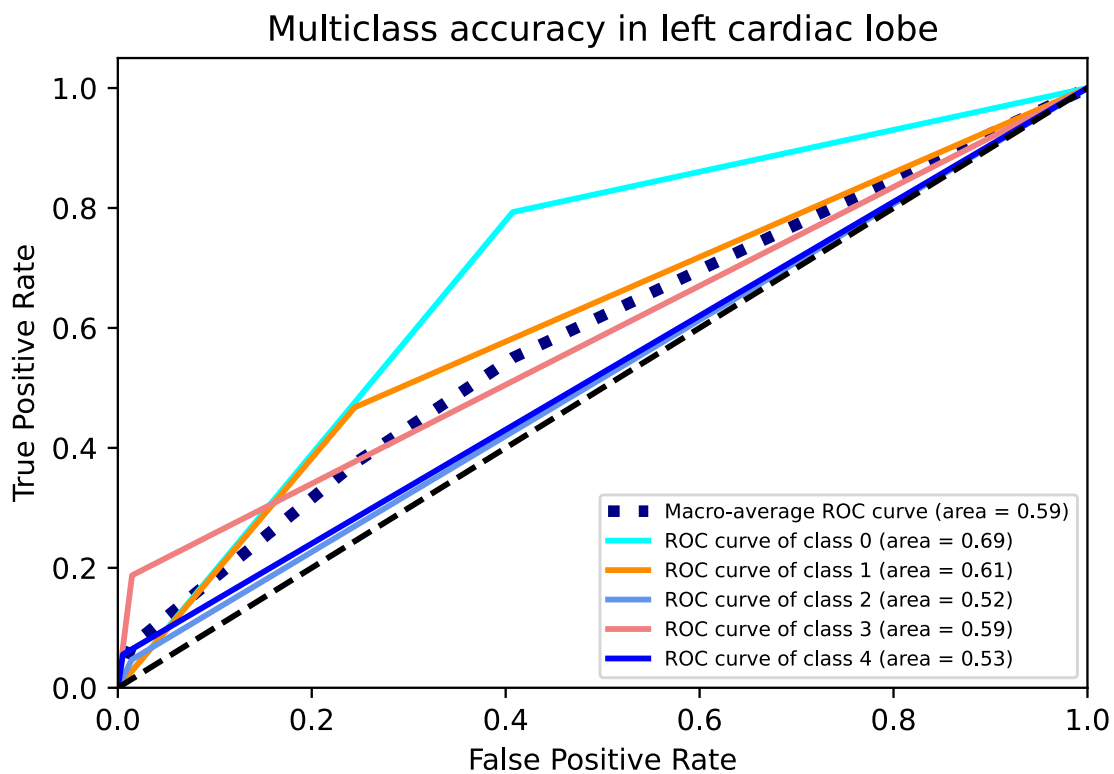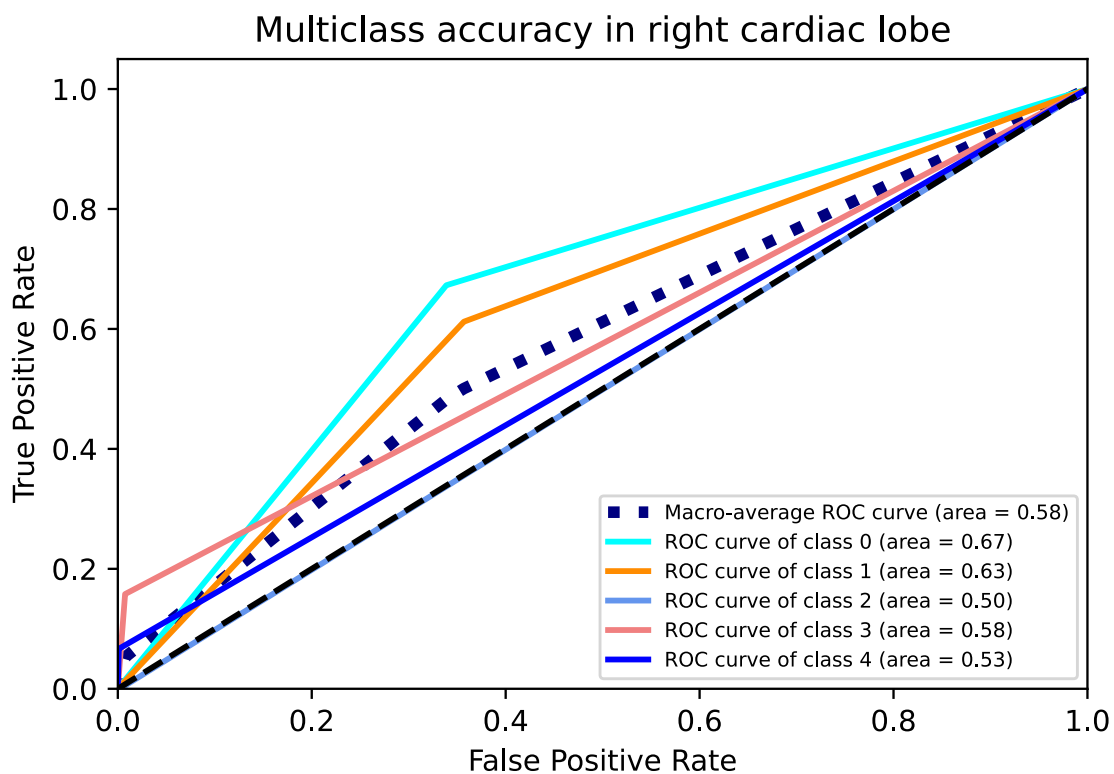

Supplement: Supplementary file 2 — Additional file 2: Multiclass accuracy for the computer vision system in the left and right cardiac lobes. Classes are based on Madec and Kobisch [30] [file 13567_2024_1432_MOESM2_ESM.pdf]

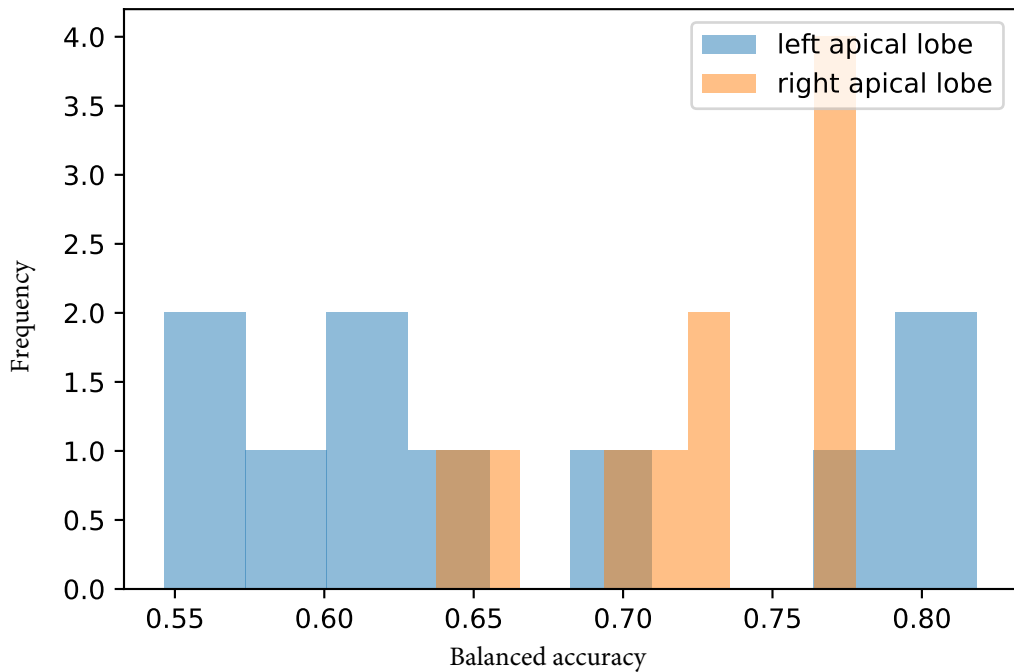

Supplement: Supplementary file 7 — Additional file 7: Distribution of the balanced accuracy between pairs of evaluators in the binary setting. Data is shown for the left and right apical lobes. [file 13567_2024_1432_MOESM7_ESM.pdf]

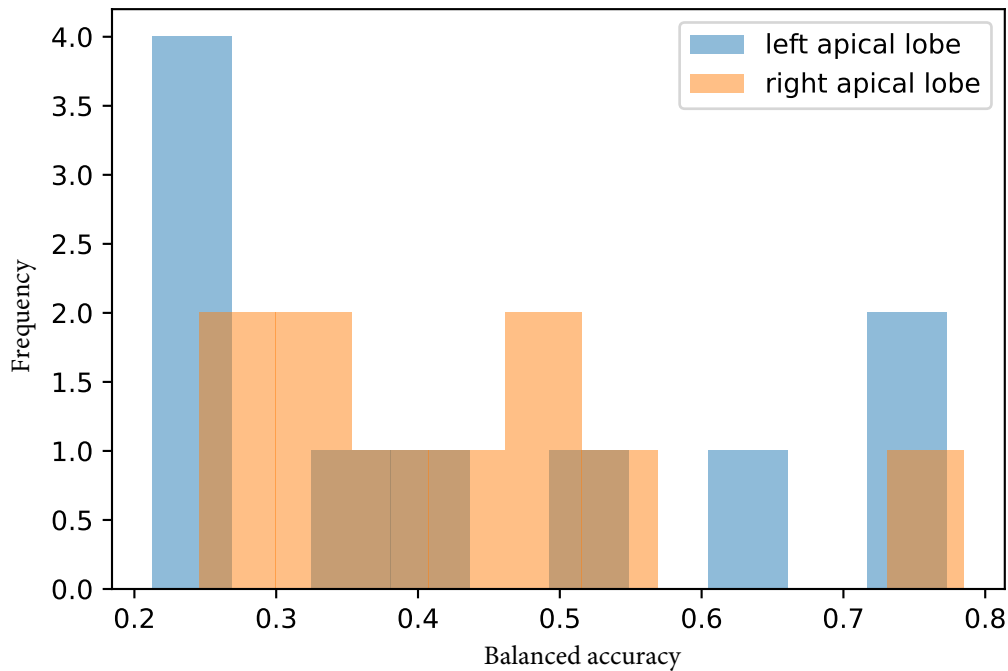

Supplement: Supplementary file 8 — Additional file 8: Distribution of the balanced accuracy between pairs of evaluators in the multiclass setting. Data is shown for the left and right apical lobes. Classes are based on Madec and Kobisch [30]. [file 13567_2024_1432_MOESM8_ESM.pdf]
