# Supplementary material for: Scoring of swine lung images: a comparison between a computer vision system and human evaluators
Source: Vet Res. 2025 Jan 13;56:9. doi: 10.1186/s13567-024-01432-5 (PMC11731141; doi:10.1186/s13567-024-01432-5)
Supplement: Supplementary file 3 — Additional file 3: Multiclass accuracy for the computer vision system in the left and right diaphragmatic lobes. Classes are based on Madec and Kobisch [30]. [file 13567_2024_1432_MOESM3_ESM.pdf]

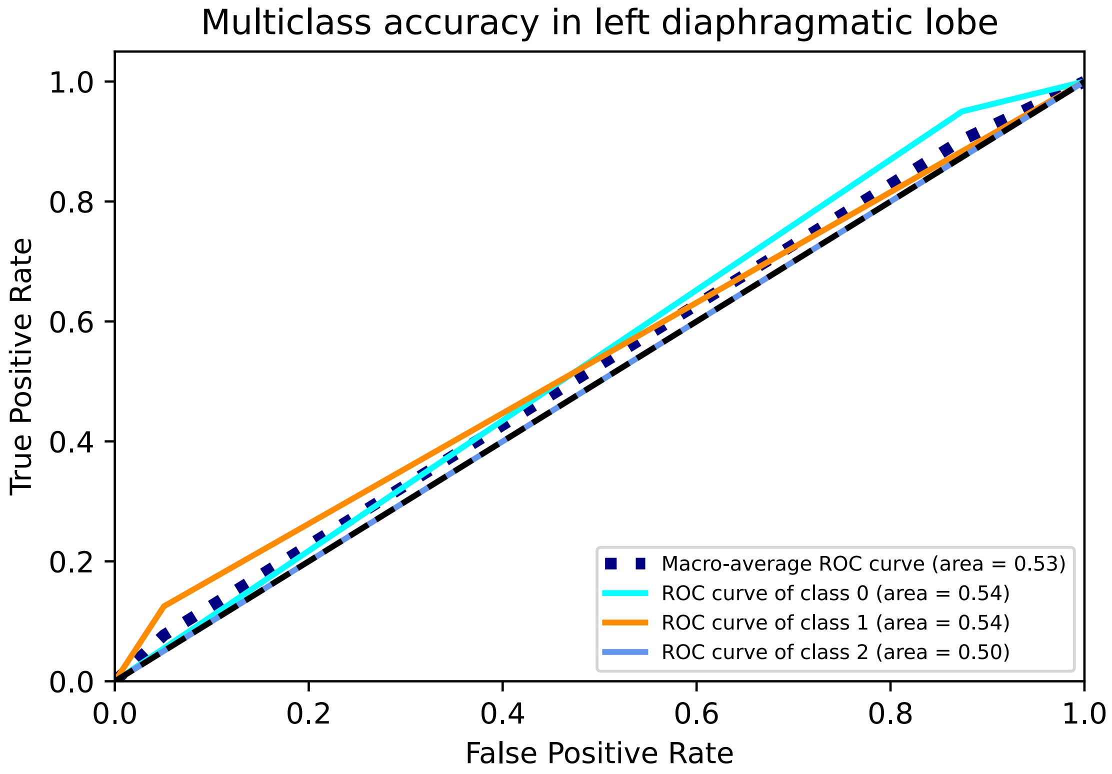
Multiclass accuracy in left diaphragmatic lobe
True Positive Rate
False Positive Rate
0.0
0.2
0.4
0.6
0.8
1.0
Macro-average ROC curve (area = 0.53)
ROC curve of class 0 (area = 0.54)
ROC curve of class 1 (area = 0.54)
ROC curve of class 2 (area = 0.50)

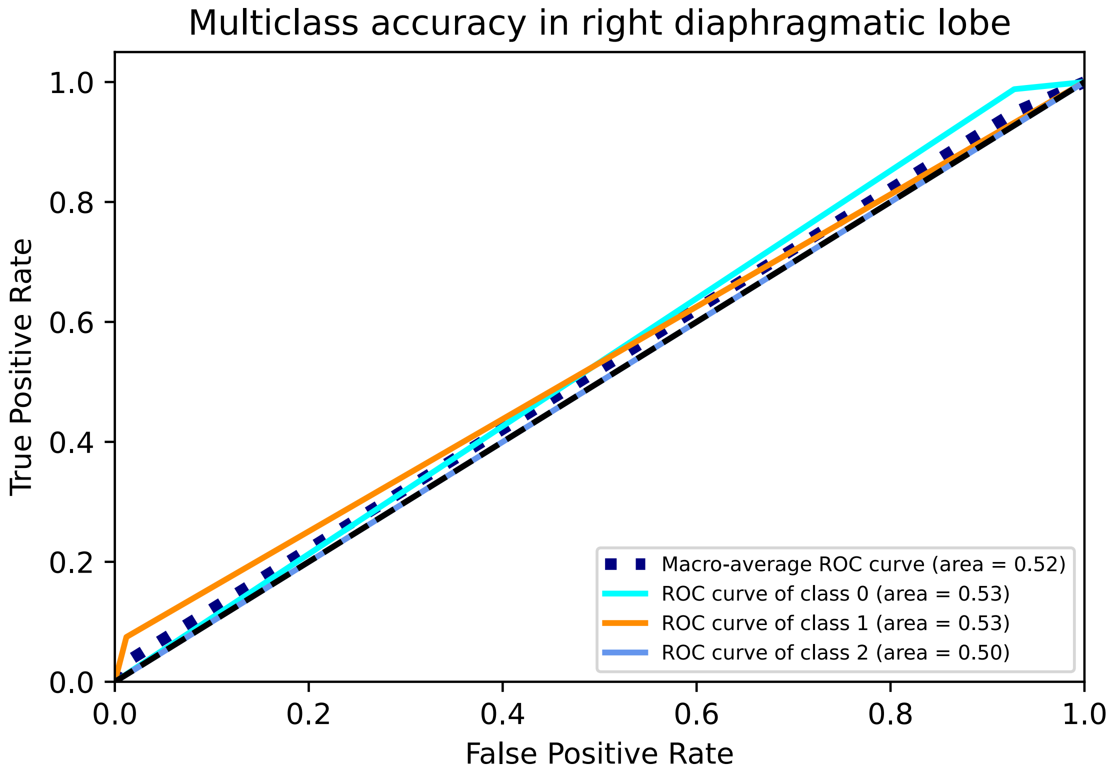
Multiclass accuracy in right diaphragmatic lobe
True Positive Rate
False Positive Rate
0.0
0.2
0.4
0.6
0.8
1.0
Macro-average ROC curve (area = 0.52)
ROC curve of class 0 (area = 0.53)
ROC curve of class 1 (area = 0.53)
ROC curve of class 2 (area = 0.50)
