# Supplementary material for: Scoring of swine lung images: a comparison between a computer vision system and human evaluators
Source: Vet Res. 2025 Jan 13;56:9. doi: 10.1186/s13567-024-01432-5 (PMC11731141; doi:10.1186/s13567-024-01432-5)
Supplement: Supplementary file 4 — Additional file 4: Binary accuracy for the computer vision system in the left and right apical lobes. [file 13567_2024_1432_MOESM4_ESM.pdf]

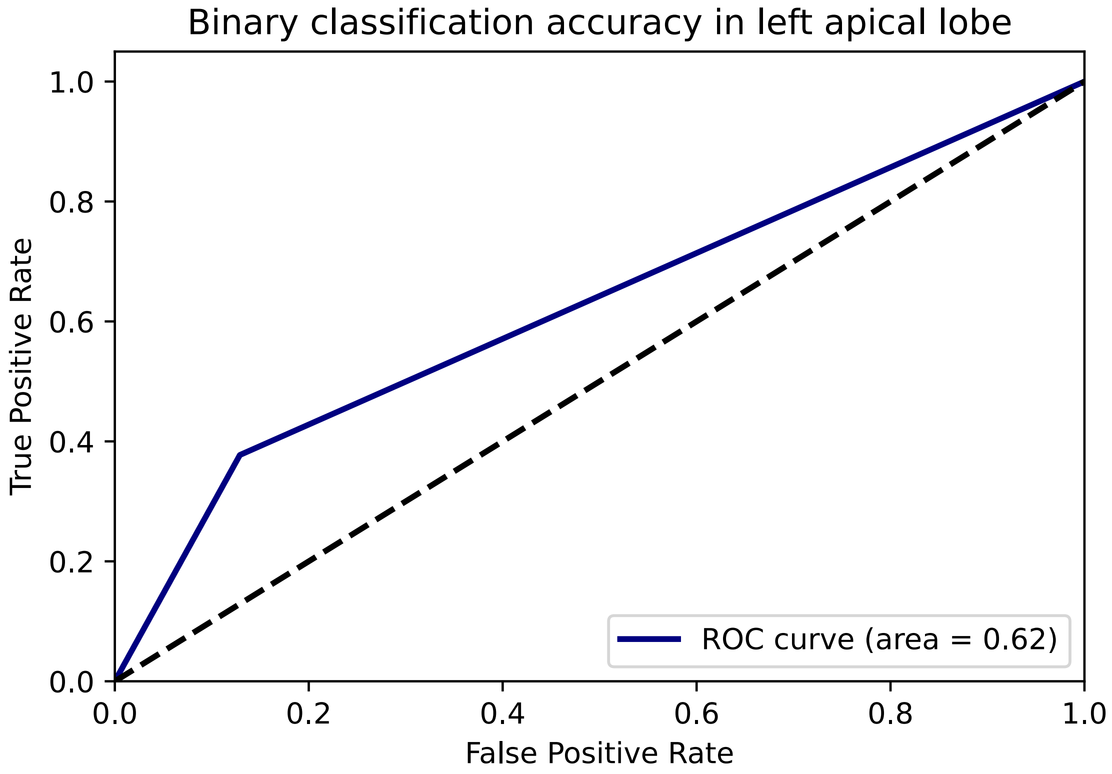

Binary classification accuracy in left apical lobe
True Positive Rate
False Positive Rate
ROC curve (area = 0.62)
0.0
0.2
0.4
0.6
0.8
1.0

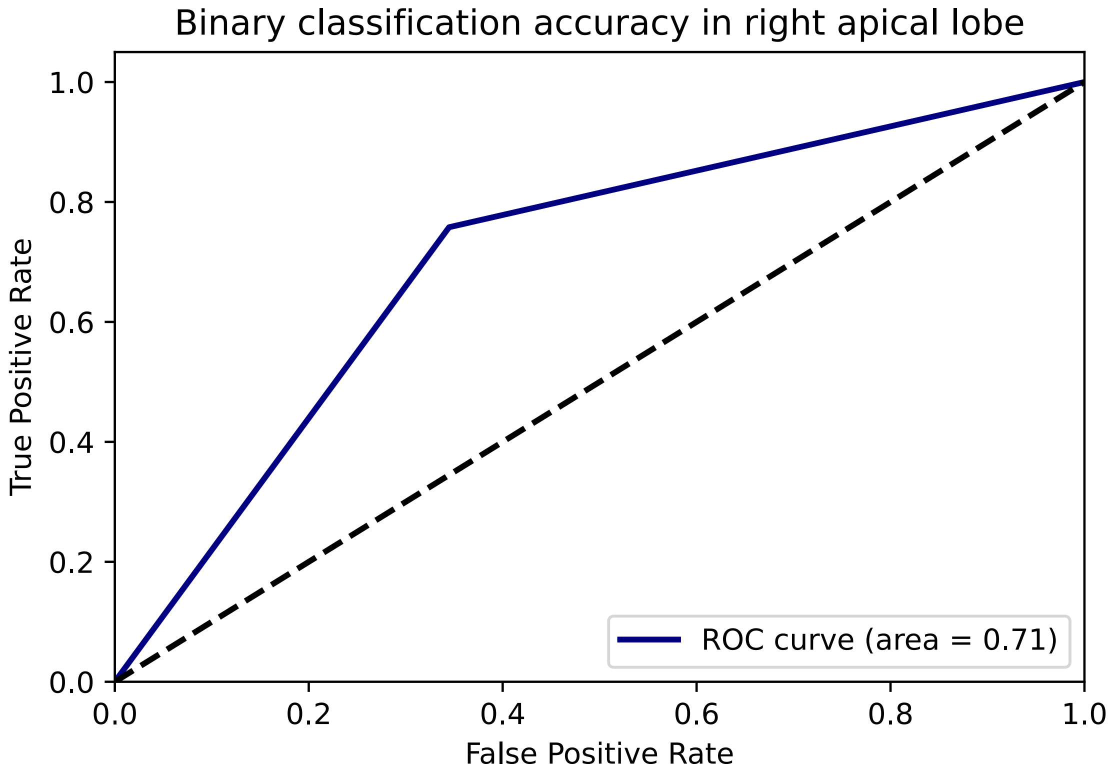

Binary classification accuracy in right apical lobe
True Positive Rate
False Positive Rate
ROC curve (area = 0.71)
0.0
0.2
0.4
0.6
0.8
1.0
