# Supplementary material for: Scoring of swine lung images: a comparison between a computer vision system and human evaluators
Source: Vet Res. 2025 Jan 13;56:9. doi: 10.1186/s13567-024-01432-5 (PMC11731141; doi:10.1186/s13567-024-01432-5)
Supplement: Supplementary file 6 — Additional file 6: Binary accuracy for the computer vision system in the left and right diaphragmatic lobes. [file 13567_2024_1432_MOESM6_ESM.pdf]

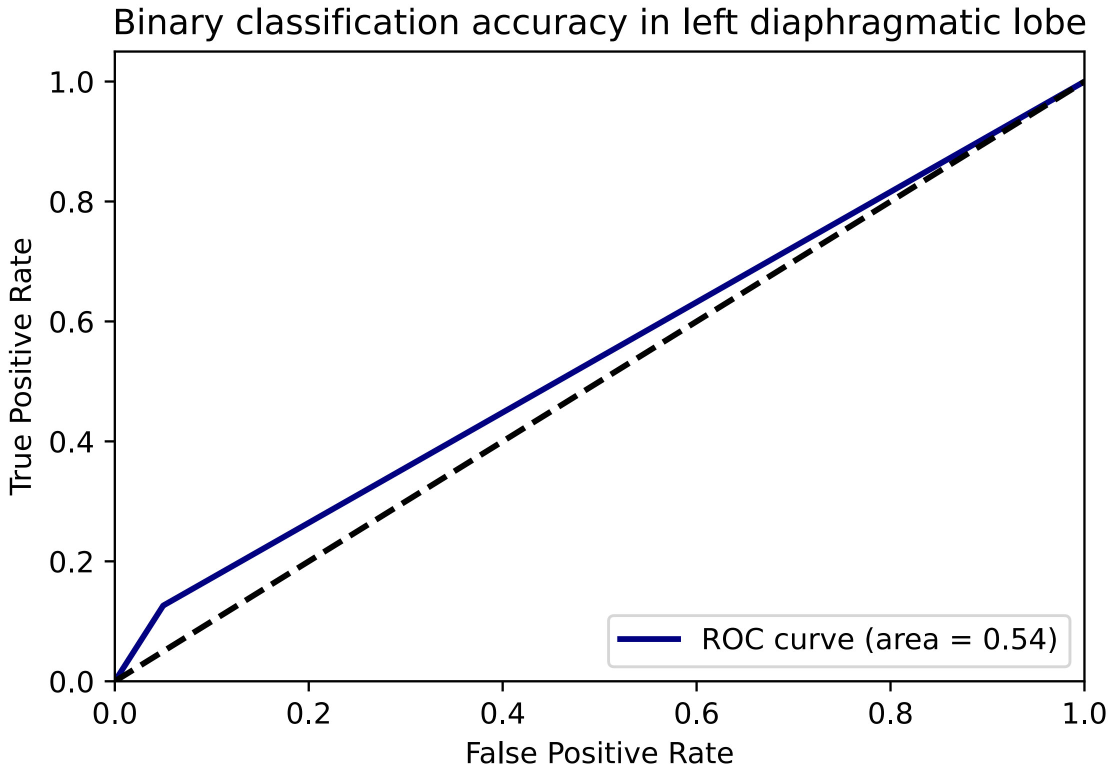
Binary classification accuracy in left diaphragmatic lobe
True Positive Rate
False Positive Rate
ROC curve (area = 0.54)
0.0
0.2
0.4
0.6
0.8
1.0

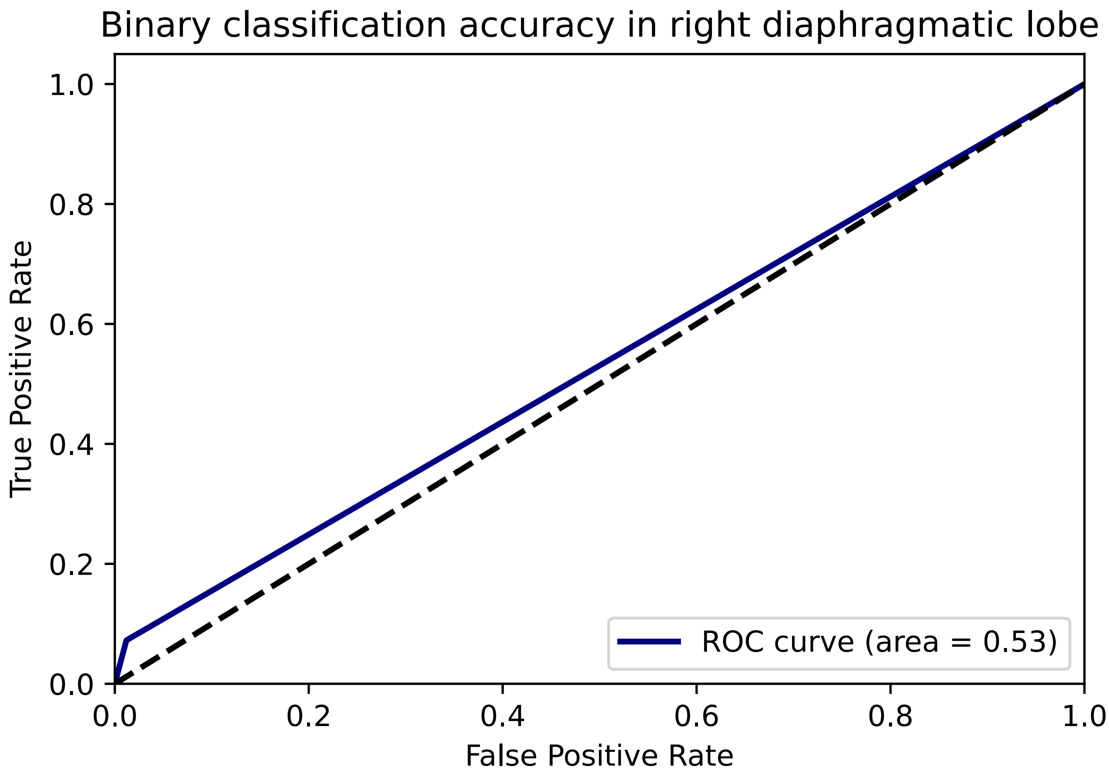
Binary classification accuracy in right diaphragmatic lobe
True Positive Rate
False Positive Rate
ROC curve (area = 0.53)
0.0
0.2
0.4
0.6
0.8
1.0
